# Parallel Profiling of Fission Yeast Deletion Mutants for Proliferation and for Lifespan During Long-Term Quiescence

**DOI:** 10.1534/g3.114.014415

**Published:** 2014-12-01

**Authors:** Theodora Sideri, Charalampos Rallis, Danny A. Bitton, Bruno M. Lages, Fang Suo, María Rodríguez-López, Li-Lin Du, Jürg Bähler

**Affiliations:** * University College London, Research Department of Genetics, Evolution & Environment and Institute of Healthy Aging, Gower Street, Darwin Building, London WC1E 6BT, UK; † National Institute of Biological Sciences, Beijing 102206, China

**Keywords:** cellular aging, longevity, quiescence, G0 phase, competitive growth

## Abstract

Genetic factors underlying aging are remarkably conserved from yeast to human. The fission yeast *Schizosaccharomyces pombe* is an emerging genetic model to analyze cellular aging. Chronological lifespan (CLS) has been studied in stationary-phase yeast cells depleted for glucose, which only survive for a few days. Here, we analyzed CLS in quiescent *S. pombe* cells deprived of nitrogen, which arrest in a differentiated, G0-like state and survive for more than 2 months. We applied parallel mutant phenotyping by barcode sequencing (Bar-seq) to assay pooled haploid deletion mutants as they aged together during long-term quiescence. As expected, mutants with defects in autophagy or quiescence were under-represented or not detected. Lifespan scores could be calculated for 1199 mutants. We focus the discussion on the 48 most long-lived mutants, including both known aging genes in other model systems and genes not previously implicated in aging. Genes encoding membrane proteins were particularly prominent as pro-aging factors. We independently verified the extended CLS in individual assays for 30 selected mutants, showing the efficacy of the screen. We also applied Bar-seq to profile all pooled deletion mutants for proliferation under a standard growth condition. Unlike for stationary-phase cells, no inverse correlation between growth and CLS of quiescent cells was evident. These screens provide a rich resource for further studies, and they suggest that the quiescence model can provide unique, complementary insights into cellular aging.

Aging is the major risk factor for complex pathologies such as cancer, cardiovascular disease, diabetes, and neurodegeneration. Model organisms have been used extensively to study the genetic basis of aging, and several aging-related processes are remarkably conserved from yeast to human (Lopez-Otin *et al.* 2013). Since the first discovery of lifespan-extending mutations in worms (Kenyon *et al.* 1993), numerous genes have been uncovered that positively or negatively affect longevity in various model systems (Fontana *et al.* 2010; Gems and Partridge 2013)

Chronological lifespan (CLS), defined as the time cells survive in a nondividing state, has been useful to study cellular aging in the budding yeast, *Saccharomyces cerevisiae* (Longo *et al.* 2012). To this end, researchers typically assay survival during stationary phase after exhaustion of glucose, and several genome-wide screens for *S. cerevisiae* CLS mutants have been performed (Powers *et al.* 2006; Fabrizio *et al.* 2010; Matecic *et al.* 2010). The distantly related fission yeast, *Schizosaccharomyces pombe*, provides an emerging, complementary model for cellular aging. In *S. pombe*, chronological aging has also been studied in stationary-phase cultures limited by glucose, a condition in which cells mostly arrest in G2 phase and die within a few days; several genetic and environmental factors affecting CLS during stationary phase have been reported (Roux *et al.* 2006; Roux *et al.* 2009). Three aging screens have been performed in *S. pombe*: a chemical screen has uncovered compounds that extend CLS (Stephan *et al.* 2013); a genetic screen has identified four genes whose overexpression results in extension of CLS (Roux *et al.* 2010); and another genetic screen has identified deletion mutants resistant to TORC1-dependent growth inhibition, which included 26 mutants with altered CLS (Rallis *et al.* 2014).

When *S. pombe* cells are deprived of nitrogen in the absence of any mating partner, they reversibly arrest in a differentiated G0-like state, called quiescence (Yanagida 2009; Marguerat *et al.* 2012; Sajiki *et al.* 2009; Takeda *et al.* 2010). The Yanagida laboratory has pioneered studies of quiescent *S. pombe* cells, including genetic analyses of quiescence entry, short-term maintenance, and exit; unlike stationary-phase cells limited for glucose, quiescent cells remain metabolically active by recycling nitrogen and can survive for several weeks if glucose remains available (Shimanuki *et al.* 2013; Yanagida 2009). Such quiescent cells are thus physiologically adapted for long-term survival and may therefore provide a distinct, complementary model system to study chronological aging.

Here, we apply Barcode sequencing (Bar-seq) (Smith *et al.* 2009; Han *et al.* 2010) to analyze the lifespans of 2847 haploid prototroph gene deletion mutants in *S. pombe* (77.7% of all nonessential deletion mutants) (Kim *et al.* 2010), because they age together in a pool in a quiescent state without nitrogen. We provide CLS data for both wild-type and mutant strains during long-term quiescence. We focus on mutants with longer CLS than wild-type and independently verify 30 of those mutants. Using Bar-seq, we also profile the proliferation of the deletion mutants growing competitively in a pool and explore the relationship between growth and lifespan.

## Materials and Methods

### Construction of prototroph deletion strain library

The auxotrophic *ade6-M216* (or *ade6-M210*) *ura4-D18 leu1-32* markers of the Bioneer deletion library (Kim *et al.* 2010) rendered it unsuitable to screen for CLS under nitrogen-depleted conditions. We therefore applied the principle of *S. pombe* SGA (Baryshnikova *et al.* 2010) to cross out all auxotrophic markers from the Bioneer v2.0 library; thus, we obtained a prototroph deletion library. To this end, the haploid v2.0 deletion mutants were crossed with the 972 *h^−^* strain on SPA plates and left to sporulate at 25° for 2 d. The plates were transferred to 42° for 3 d to eliminate vegetative cells. Spores were then transferred to yeast extract with supplements (YES) medium and left to germinate for 2 d. The library was then successively spotted on Edinburgh minimal medium (EMM; Formedium) to select for prototrophs and on YES medium with G418 to select for the kanMX4 cassette used for generating deletions. Altogether, we performed three rounds of EMM and YES+G418 selection. Because *leu1-32* is strongly linked to the mating-type locus, the selection for prototrophs also led to a selection for *h^−^* strains. We estimated that <1% of the segregants were *h^+^* cells, based on quantitations of mating frequency in the mutant pool. In the process of converting the library to prototrophs, some mutants have been lost due to genetic linkage. We therefore ended up with 2847 mutants in the prototroph pool instead of 3004 in the original library

### Generation of pooled deletion strain library

Using the RoToR robot (Singer Instruments), we compacted the prototroph deletion library into 9 plates at 384 colonies per plate. Strains were grown for 2 d on YES plates containing ampicillin, kanamycin and G418. Colonies were then washed off the plates with 5 ml of 20% (v/v) glycerol in YES per plate, pooled together, aliquoted, and stored at −80°. Pooled library cultures grown at 32° to OD_600_ 0.6 in EMM without nitrogen were tested for spores. To this end, 5% (w/v) SDS was added to the cells following incubation at 30° for 30 min before plating onto YES agar plates; growth was not observed after 3 d of incubation at 32°. This result indicated absence of spores, reflecting that the pool almost exclusively consists of *h^−^* cells (see above).

We examined the prototroph mutant pool using FACS and visual analyses of proliferating mutant cells: in both EMM and YES media the cells were mainly in G2 phase, and there was no indication of diploid cells (data not shown). Some auxotroph mutants were included in the supposedly prototroph library. We therefore examined the original (not prototroph) deletion mutants of *lys7*, *his1*, *ura1*, *ade9* whose barcodes were detected in the prototroph pool. These deletion strains did not grow when streaked on corresponding selective plates, suggesting that these deletion mutants are auxotrophic. However, when using the RoToR for plating (as used when making the prototroph library), some residual growth of the prototroph mutants was evident on selective media. This result suggests that when a cell mass is printed onto selective media (without corresponding supplements, but not in the case for antibiotics), residual growth can lead to a slight increase in biomass. This phenomenon suggests a community effect with nutrient sharing or colony compartmentalization as seen for budding yeast (Cap *et al.* 2012). Alternatively, or in addition, some auxotrophic mutants might be leaky and allow some growth, especially when plated using the RoToR.

### Chronological lifespan assays during long-term quiescence

We thawed 250 μl of the prototroph library pool, inoculated in 250 ml YES medium, and left it at 25° for 16 hr without shaking. We then centrifuged 50 ml from the culture at 420×*g* for 2 min, washed the cell pellet twice with 50 ml EMM, resuspended the cells in 250 ml EMM, and grew them overnight at 30° with shaking (130 rpm) up to OD_600_ ∼0.5. The cells were then collected by centrifugation at 420×*g* for 2 min, washed twice in an equivalent volume of pre-warmed EMM without nitrogen (EMM-N), and resuspended in 800 ml EMM-N. The OD_600_ was adjusted to 0.15, and the cells were grown for 2 d at 32° with shaking (130 rpm) to a final OD_600_ ∼0.6. This condition was used as the reference timepoint (t = 0) after cells stopped to proliferate. We then collected 50–100 ml of cell culture once per week (until viability was <10%) by centrifugation at 960×*g* for 3 min, one wash in 0.5 ml EMM-N, and resuspension in 0.5 ml EMM-N+0.5 ml 50% (v/v) glycerol. Cells were then stored at −80° until use for DNA extraction and generation of the Bar-seq libraries.

The medium of the quiescent cultures was replaced twice per week for the first 4 wk, and once per week thereafter to avoid depletion of glucose and other nutrients as well as accumulation of toxic by-products from metabolism. When initially replacing the medium less than twice per week, cell viability did decrease to ∼40–50% within 4 wk. To determine cell viability during long-term quiescence, cells were counted using a Beckman Coulter counter, diluted to ∼200 cells per 100 μl of YES, and 100 μl of diluted cells were plated on 3 YES plates each (300 μl total). After 3 d of incubation at 32°, the colonies on the plates were counted and viability was calculated for each timepoint relative to the reference timepoint. Two independent biological replicates of the deletion pool and individual strains were assayed.

To determine chronological lifespans of individual mutants compared with wild-type, 40 ml cultures were prepared in EMM-N as described. Samples were taken at weekly intervals, serially diluted (OD_600_ 0.1, 0.01, 0.001), and 5 µl of diluted cell suspensions were spotted onto YES plates. Cells were left to grow at 32° for 3 d before photos were taken.

### Re-growth of quiescent cells

For the re-growth CLS screen, 100 µl aliquots from quiescent cells stored at −80° in glycerol were spread on YES plates and left to grow at 32° for 1 d (0, 4, 8 wk in quiescence) or 2 d (12, 14 wk in quiescence) at 32°. Cells were scraped from the agar with 1 ml YES and the suspension was transferred to a 1.5-ml tube and centrifuged at 960×*g* for 1 min. These cell pellets were used for DNA extraction and barcode sequencing.

### Genomic DNA extraction and Bar-seq library preparation

The MasterPure Yeast DNA purification kit (Epicentre) was used for DNA extraction. Barcodes were amplified with Ex Taq DNA polymerase (TaKaRa) using two rounds of PCR as previously described (Han *et al.* 2010). The following primers were used for the first round of PCR. For amplification of uptags, forward primer 5′-CACGACGCTCTTCCGATCTxxxxGAGGCAAGCTAAGATATC-3′ and reverse 5′-AGCAGAAGACGGCATACGAGATATTGGCGTGACTAGTTCAGACGTGTGCTCTTCCGATCT GCCTTACTTCGCATTTA-3′ reverse. For amplification of downtags, forward 5′- CACGACGCTCTTCCGATCTxxxxCCAGTGTCGAAAAGTATC-3′ and reverse primer 5′-AGCAGAAGACGGCATACGAGATATTGGCGTGACTGGAGTTCAGACGTGTGCTCTTCCGATCTTTGCGTTGCGTAGG-3′. The xxxx within the sequence represents four unique nucleotide indexes that allowed us to multiplex for sequencing (index sequences available on request). We used 10 pmol per primer for each reaction and the following program for the thermocycler: 94°/4 min, (94°/20 sec, 53°/20 sec, 72°/20 sec) × 30 cycles, 72°/20 sec. For the second round of PCR, the forward primer was 5′- AATGATACGGCGACCACCGAGATCTACACTCTTTCCCTACACGACGCTCTTCCGATCT-3′ and the reverse primers was 5′-AGCAGAAGACGGCATACGAGATATTGGCGTGACTGGAGTTCAGACGTGTGCTCTTCCGATCT-3′. The program used for the thermocycler for the second round of PCR was as follows: 94°/4 min, (94°/20 sec, 56°/20 sec, 72°/20 sec) × 20 cycles, 72°/20 sec. The PCR products were run on 1.5% (w/v) agarose gels, and the bands were excised and purified using the QIAquick gel extraction kit (Qiagen). The amplified and purified barcodes were quantified using the Qubit Fluorometer (Invitrogen) and Bioanalyzer (Agilent). The samples were then mixed together in equal molar ratios and used for Illumina sequencing using either MiSeq (MiSeq v2 Reagent kit) or HiSequation 2000 (for re-growth screen) instruments. Fifty cycles of single-end sequencing were used, with 20 multiplexed samples for each library.

### Barcode decoding

The barcode sequences of the Bioneer v2 library were experimentally characterized by high-throughput sequencing. Using a primer extension procedure used previously for paired-end-sequencing-based decoding (Han *et al.* 2010), we amplified the barcode sequences together with the flanking genomic DNA sequences from the genomic DNA of pooled mutants. The PCR products were single-end sequenced on a MiSeq instrument for 101 cycles. Each MiSeq sequencing read contains a 4-bp multiplexing index, 18-bp PCR primer sequence, 20-bp barcode, 26-bp universal spacer, and 33-bp flanking genomic sequence. After grouping reads with the same barcode sequence together, their flanking genomic sequences were compared with the expected flanking sequences obtained from the file http://pombe.kaist.ac.kr/nbtsupp/download/All_primers%20used_in_gene%20deletion.xls (Kim *et al.* 2010). Our analysis identified barcodes for 2473 genes. Among them, 1871 genes have both uptag and dntag decoded, 254 genes have only uptag decoded, and 348 genes have only dntag decoded. The decoded barcodes are listed in Supporting Information, Table S1 and used in the data analysis.

### Data analysis

The decoded barcodes for Bioneer v2 were combined with the barcodes for Bioneer v1 (Han *et al.* 2010) to maximize the set of mutants that can be interrogated. Uptags and dntags, together with universal primers and 20 multiplex index sequences, were used to construct a FASTA database, in which each gene had 20 sequence entries of exactly 51 bp (multiplex index, 4 bp; universal primer, 18 bp; up/downtag, 20 bp; universal primer, 9 bp). This database contains 98,280 sequence entries and is available on request. To identify and quantify sequence reads per mutant in a given sample, raw sequence data were aligned against the database using Bowtie 0.12.7 (Langmead *et al.* 2009); only a single mismatch within the first 42 bp were tolerated, and only unique matches across the entire database were retained. Two independent biological replicates were performed, exploiting the variation to assess statistical significance (Robinson *et al.* 2014). Timepoints for which we obtained less than 10 reads were excluded from further analysis. The number of reads for each mutant in a timepoint “i” was normalized for the sequencing depth of the same timepoint (reads per mutant_i_/depth_i_). Fold changes relative to the reference timepoint (reads per mutant_0_/depth_0_) were determined. To identify CLS mutants, a lifespan score was calculated. The average of weighted up-tags and down-tags for each timepoint was calculated as previously described (Han *et al.* 2010; Sun *et al.* 2013). To weight a barcode at a specific timepoint i, we determined the sum of reads for that barcode at timepoint i and reference (timepoint 0) divided by the sum of reads of both barcodes at timepoints 0 and i. For example, for the uptag at timepoint 8 wk, the weighted value was: (Up_8_+Up_0_)/(Up_8_+Dn_8_+Up_0_+Dn_0_). Then, the median of all timepoints (except t = 0, reference) was determined and the average for the two biological replicates was defined as the lifespan score. The lifespan score represents the median of all fold-changes throughout the time course for the two replicates. Growth scores were calculated as follows. The number of reads for each mutant in a timepoint “i” was normalized for the sequencing depth of the same sample (reads per mutant_i_/depth_i_). Fold changes relative to the reference timepoint at 120 min (reads per mutant_120_/depth_120_) were then determined. The average of weighted up-tags and down-tags for each timepoint was calculated as described (Han *et al.* 2010; Sun *et al.* 2013), and the median of all timepoints, except t = 120 min (reference timepoint), was determined. Then, the average of both biological repeats was determined and defined as the growth score, which thus represents the median of all fold-changes throughout the time course.

### Classification and verification of mutants

Genetic interactions were analyzed using the *S. pombe* genetic interactome tool (Ryan *et al.* 2012). Diagnostic PCRs for both deletion junctions were performed to confirm deletion of the correct gene for 30 random deletion mutants that showed CLS phenotypes. PCR primer sequences were as reported (Kim *et al.* 2010). The MasterPure Yeast DNA purification kit (Epicentre) was used for DNA extraction and the following program for the thermocycler: 94°/2 min, (94°/30 sec, 53°/30 sec, 72°/90 sec) × 30 cycles, 72°/7 min.

## Results and Discussion

### Lifespan of wild-type cells during quiescence in absence of nitrogen

**Figure 1:**
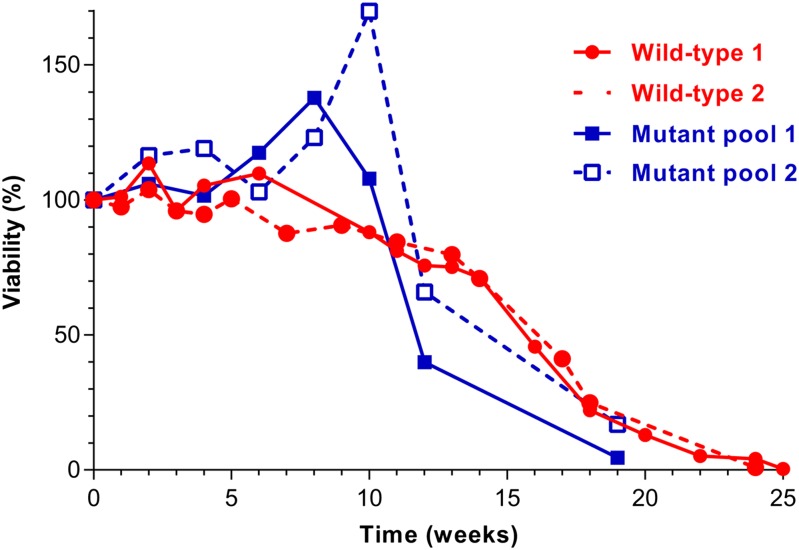
CLS assays during long-term quiescence under nitrogen deprivation. Two independent biological repeats each for wild-type and for deletion mutant pool were performed. Viability was determined from colony-forming units (CFUs) on plated cells. The CFUs determined at timepoint 0 was defined as 100% survival, and survival at the other timepoints was determined relative to this reference timepoint. For the Bar-seq screen, the mutant-pool experiments shown here were used. The timepoints at which pooled mutants were harvested for Bar-seq analysis are indicated in blue on the X-axis.

We first determined the CLS of wild-type fission yeast during long-term quiescence in the absence of nitrogen by determining cell viability in weekly intervals (Figure 1, red curves). Cell viability remained above 70% for approximately 14 wk, followed by a stronger decrease over the next month, but only after 24–25 weeks did viability decrease below 1%. The median CLS was approximately 16.6 wk (polynomial regression/order 2). This long CLS is in stark contrast to stationary-phase fission yeast cells limited by glucose, which survive for less than 1 wk under standard conditions and up to ∼18 days with low initial glucose concentration (Roux *et al.* 2009). This difference indicates that fission yeast can cope much better with nitrogen deprivation than with glucose deprivation, which might suggest that the ecological conditions encountered by wild strains are not normally limited for glucose. It has been proposed that the natural environment of both fission and budding yeasts contains abundant carbon sources but variable nitrogen sources (Weisman 2010; Backhus *et al.* 2001). Moreover, the CLS determined here is much longer than for quiescent budding yeast cells in the absence of nitrogen, which show greatly reduced viability after 12 d (Dziedzic and Caplan 2012). We therefore propose that long-term quiescence of fission yeast cells can serve as a complementary condition to study chronological aging. This condition could provide insight into aging of terminally differentiated postmitotic cells or for stem cells that alternate between proliferating and quiescent states.

### Lifespan of prototrophic deletion pool during quiescence

The Bioneer v2.0 haploid deletion library contains 3004 nonessential mutants in an auxotroph *ade6-M216 leu1-32 ura4-D18* background (Kim *et al.* 2010). These mutants therefore need to be grown in medium supplemented with adenine, leucine, and uracil, which would provide a nitrogen source during long-term quiescence. To screen for deletion mutants with altered CLS in the absence of nitrogen, we therefore needed to cross out the auxotrophic markers from all deletion mutants to generate a prototroph deletion library (see *Materials and Methods*). This back-crossing of all mutants to a wild-type strain also tested for co-segregation of any phenotypes with the deletion marker. All prototroph mutants were then pooled together in preparation for the parallel screening.

We next monitored overall CLS of the prototrophic deletion library pool during quiescence (Figure 1, blue curves). Cell viability remained at higher levels than for wild-type cells for approximately 10 wk, transiently even surpassing the 100% initial viability of the reference timepoint, followed by a stronger decrease in viability than for wild-type over the next 9 wk. These differences compared with the CLS profiles of wild-type cells could be expected as different mutants will lose viability at different rates in the pool. The transient increase in viability of the pool could reflect that nitrogen sources released from dead mutants allow other mutants to temporarily proliferate again.

### Bar-seq screen for deletion mutants with altered lifespans during quiescence

We next screened the 2847 prototrophic deletion mutants in parallel for altered CLS during quiescence in the absence of nitrogen. Using the two independent biological repeats of the mutant pool quiescence experiments shown in Figure 1, we collected samples at timepoints 0, 4, 8, 12, and 14 wk. These samples were then further processed for Bar-seq analysis (see *Materials and Methods*). This approach takes advantage of the two unique barcode sequences associated with each deletion mutant, called uptag and downtag (Kim *et al.* 2010). Changes in relative abundance of the mutants as a function of chronological aging can thus be detected by next-generation sequencing of these barcodes. Mutants with shorter CLS than the pool average will become under-enriched, whereas those with longer CLS will become enriched with time relative to the reference timepoint at the beginning of the time course. Such parallel quantitative phenotyping is highly sensitive and efficient (Han *et al.* 2010; Sun *et al.* 2013). Barcode decoding for v2 Bioneer was performed in this study (see *Materials and Methods*), and the barcodes are provided in Table S1.

In studies reporting CLS screens for budding yeast mutants, aging cells have been re-grown before DNA extraction to avoid any noise from dead cells (Fabrizio *et al.* 2010; Matecic *et al.* 2010). Here, as our main approach, we chose to directly extract DNA from quiescent cells rather than re-growing them beforehand. This approach has the advantage that the measured proportion of the mutants represents their proportion in the pool at time of sample collection, without any bias being introduced by differences in quiescence exit or growth rate among the mutants. DNA is expected to become rapidly degraded on cell death and therefore should not substantially affect the assay. Moreover, it is less likely that DNA from nonlysed dead cells was present in our samples, because the time interval between timepoints was 2–4 wk rather than a few days as in the budding yeast studies. Nevertheless, we also performed one CLS screen (two biological replicates) with cells that were re-grown before DNA extraction and Bar-seq analysis. The two complementary approaches showed good agreement (see below).

Of the 2847 prototroph mutants, we detected 75% and 65% with ≥1 or ≥10 sequence reads, respectively, at timepoint zero for each of the replicates. Seventy percent of sequencing reads could be mapped to the decoded barcodes (10,555,488 reads out of a total of 15,087,758 processed reads over all 20 samples, including 5 timepoints, 2 tags, and 2 repeats). The deletion junction information was downloaded from the KAIST website. The barcodes that failed to be detected may have deletion junctions different from those provided by KAIST for the following reasons: the sequences could have been altered due to oligonucleotide synthesis errors or *in vivo* mutations, or some strains (especially those that have been upgraded) could have been created with primers different from the original design. Such discrepancies have also been observed when decoding the v1.0 haploid deletion library (Han *et al.* 2010).

The number of sequencing reads for the uptags always correlated with those of the downtags for all samples (*r*_Pearson_ ∼0.64–0.95). The sequencing counts for each mutant are provided in Table S2. We obtained at least one barcode read for 2563 deletion mutants and could determine reliable CLS data for 1199 of these mutants (Table S3).

Reassuringly, barcode reads for deletion mutants in genes reported to be necessary for entry into or maintenance of quiescence (Sajiki *et al.* 2009) were either absent or present at very low numbers (<10) throughout the time course (Table S2). A similar pattern was evident for all autophagy mutants; although some of these mutants were detected at the reference timepoint and in the pools of proliferating cells, the counts decreased dramatically from 4 wk onwards (Table S2). This finding was expected because autophagy plays important roles for lifespan during quiescence in *S. pombe* (Kohda *et al.* 2007) and other organisms (Markaki and Tavernarakis 2013).

**Figure 2:**
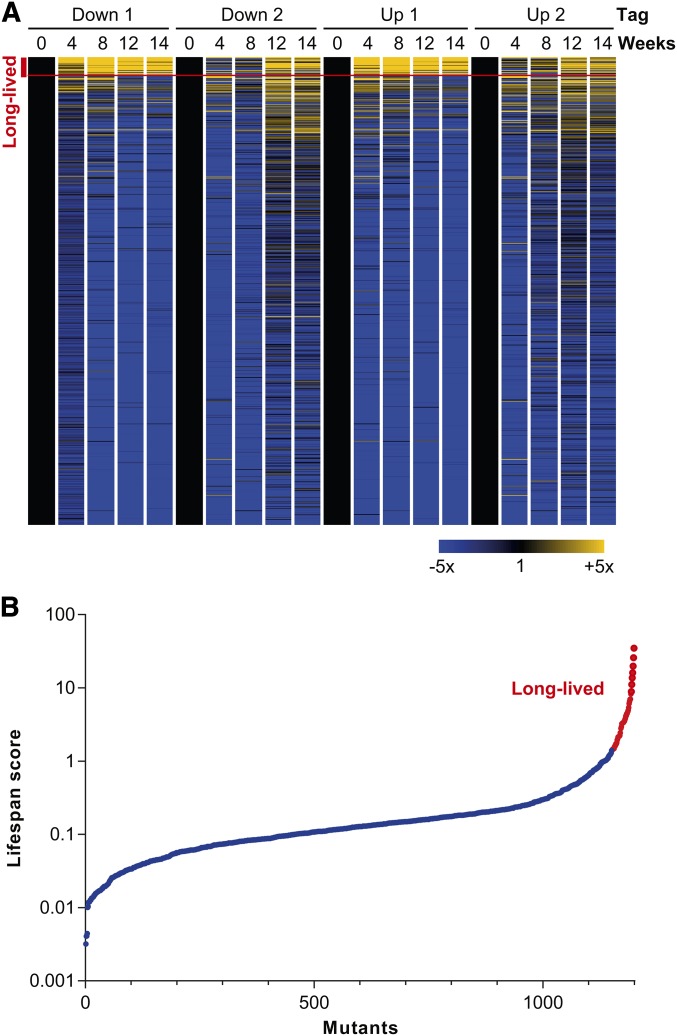
CLS profiling and lifespan scores of deletion mutants. (A) CLS profiles showing fold-changes for the two barcode tags relative to the reference timepoint (t = 0). The columns show the data for the two biological repeats for the downtags (Down 1 and 2) and uptags (Up 1 and 2) at the five timepoints analyzed (0–14 wk). The rows correspond to the 1199 deletion mutants for which lifespan scores could be determined. The fold changes in tag abundance relative to timepoint 0 are color-coded as indicated at bottom right. The mutants are ordered by lifespan scores, with highest scores at top. The 48 mutants defined as "long-lived" (Table 1) are indicated with a red bracket at top left. The heat map was generated with GeneSpring (Agilent). (B) Plot of ordered lifespan scores for 1199 deletion mutants (see *Materials and Methods*). The 48 long-lived mutants are indicated in red.

Many mutants decreased in abundance over the time course relative to the reference timepoint (Figure 2A). The 10% of mutants with the lowest lifespan scores are provided in Table S4. Several different deficiencies and indirect effects, not necessarily related to chronological aging, could underlie these phenotypes. However, the few mutants that increased over the time course relative to the reference timepoint (Figure 2A), and therefore have longer lifespans than the pool average, are more likely to directly interfere with the aging process. We therefore focused the analysis and discussion below on these long-lived mutants (Table 1). The data for the alternate CLS screen with cells re-grown before Bar-seq analysis are provided in Table S5 (sequence counts per mutant in each sample) and in Table S6 (long-lived mutants unique for re-growth screen).

**Table 1:** Long-lived mutants

Mutant	Product Description in PomBase (Wood *et al.* 2012)	Verified Single Mutants	Long-Lived in Re-growth Screen	GO Cellular Component (Blake *et al.* 2013)
SPBC1198.07c	Mannan endo-1,6-alpha-mannosidase (predicted)	Yes	Yes	Integral component of membrane, endoplasmic reticulum
SPBC14C8.15	Triglyceride lipase-cholesterol esterase (predicted)	Not tested	Yes	Integral component of membrane, Golgi apparatus
SPBC18H10.18c	Sequence orphan	Yes	Yes	Integral component of membrane
SPCC594.02c	Conserved fungal protein	Yes	Yes	Integral component of membrane, endoplasmic reticulum
SPBC30D10.09c	HVA22/TB2/DP1 family protein	Not tested	Yes	Integral component of membrane, endoplasmic reticulum
*gyp3*	GTPase activating protein Gyp3 (predicted)	Yes	Yes	Integral component of membrane
*vps66*	Acyltransferase (predicted)	Yes	Yes	Integral component of membrane, Golgi apparatus, endoplasmic reticulum
*frp2*	Ferric chelate reductase (predicted)	Yes	Yes	Plasma membrane, endoplasmic reticulum
*ncs1*	Neuronal calcium sensor-related protein Ncs1	Yes	Yes	Plasma membrane
*rho2*	Rho family GTPase Rho2	Yes	Yes	Plasma membrane
SPCC1020.08	Wybutosine biosynthesis protein Tyw1 (predicted)	Not tested	Yes	Endoplasmic reticulum
SPAC23D3.03c	GTPase-activating protein (predicted)	Yes	Yes	Golgi apparatus
*fta5*	Cell surface glycoprotein	Not tested	No	Golgi apparatus, endoplasmic reticulum
*vta1*	Vps20-associated protein Vts1 (predicted)	Yes	Yes	
SPAC8E11.05c	Conserved fungal protein	Yes	Yes	
SPACUNK4.16c	Trehalose-phosphate synthase (predicted)	Yes	Yes	
SPBC1921.04c	Dubious	Yes	Yes	
SPCC320.03	Transcription factor (predicted)	Yes	Yes	
SPCC594.01	DUF1769 family protein	Yes	Yes	
*abp2*	ARS binding protein Abp2	Not tested	Yes	
*apl1*	AP-2 adaptor complex subunit Apl1 (predicted)	Yes	Yes	
*aps2*	AP-2 adaptor complex subunit Aps2 (predicted)	Yes	Yes	
*clr4*	Histone H3 lysine methyltransferase Clr4	Not tested	No	
*cmb1*	Cytosine-mismatch binding protein 1	Not tested	No	
*cmk2*	MAPK-activated protein kinase Cmk2	Not tested	Yes	
*apl3*	AP-2 adaptor complex subunit Alp3 (predicted)	Yes	Yes	
SPBC947.09	ThiJ domain protein	Not tested	No	
*sds23*	Inducer of sexual development Sds23/Moc1	Yes	Yes	
SPCC306.11	Sequence orphan	Not tested	No	
*gpx1*	Glutathione peroxidase Gpx1	Not tested	No	
*hht1*	Histone H3 h3.1	Yes	Yes	
*mni1*	Mago Nashi interacting protein Mni1 (predicted)	No	Yes	
*mrc1*	Mediator of replication checkpoint 1	Yes	Yes	
*msh3*	MutS protein homolog 3	Yes	Yes	
*mug161*	CwfJ family protein, splicing factor (predicted)	Not tested	Yes	
*osr1*	Short chain dehydrogenase (predicted)	Not tested	No	
SPBC4B4.12c	Sequence orphan	Not tested	No	
*pek1*	MAP kinase kinase Pek1	Not tested	Yes	
*plb1*	Phospholipase B homolog Plb1	Yes	Yes	
*dad2*	DASH complex subunit	Not tested	No	
*mug80*	Cyclin Clg1 (predicted)	Not tested	Yes	
*set9*	Histone lysine methyltransferase Set9	Yes	Yes	
*spo4*	Serine/threonine protein kinase Spo4	Yes	Yes	
*tfs1*	Transcription elongation factor TFIIS	Yes	Yes	
*tif213*	Translation initiation factor eIF2 gamma subunit (predicted)	Yes	Yes	
*gsk3*	Serine/threonine protein kinase	Yes	Yes	
SPCC794.03	Amino acid permease (predicted)	Yes	Yes	
SPBC8E4.02c	Sequence orphan	Yes	Yes	

We computed a lifespan score for each mutant that represents the proportion of each mutant in the pool across the time course relative to the reference timepoint (see *Materials and Methods*). Lifespan scores could be computed for 1199 mutants; these scores are plotted in Figure 2B, with values provided in Table S3. We identified 48 long-lived mutants, defined as those with lifespan scores >1.44 (Figure 2B; Table 1). Using the same method to determine long-lived mutants, the re-growth CLS screen also identified 39 (81%) of the 48 mutants in Table 1, in addition to another 55 long-lived mutants (Table S6). The two approaches therefore showed generally good agreement. The long-lived mutants were not enriched for any Gene Ontology (GO) categories. We exploited a genetic interactome study for fission yeast (Ryan *et al.* 2012) to examine the functional relationships among the long-lived mutants. For 35 out of 48 long-lived mutants, genetic interaction data were available. Notably, 25 out of these 35 genes showed at least one genetic interaction with other genes in our list of long-lived mutants. This result indicates that the genes identified by the screen show substantial functional coherence.

### Verification of CLS mutants

**Figure 3:**
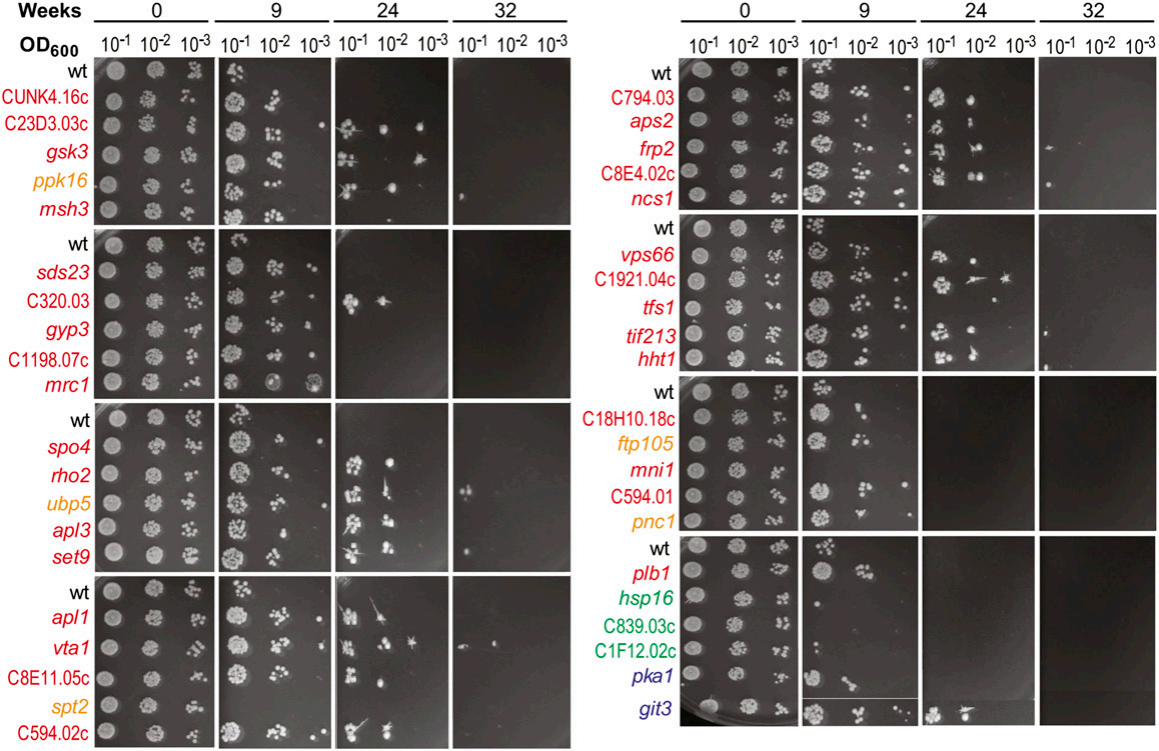
Independent verification of CLS for selected mutants in individual cultures. Mutant and wild-type (wt) control cells were assayed at 0, 9, 24, and 32 wk after entering quiescence in the absence of nitrogen. As indicated on top, 10-fold serial dilutions from each timepoint were spotted on agar plates for each culture. The mutant strains are color-coded as follows: red, long-lived mutants (Table 1); orange, mutants with high lifespan scores ∼0.4-1.2; green, lifespan score <0.14; blue, two mutants known to be long-lived during quiescence but for which no lifespan score could be computed.

We performed independent lifespan assays for selected mutants when aged as individual cultures rather than in a competitive pool. The individual mutant cells, along with wild-type reference cells, were shifted to a nitrogen-depleted minimal medium, and survival was monitored every week by spotting serial dilutions onto agar plates. We randomly selected and tested 31 out of the 48 long-lived mutants for extended CLS compared with wild-type (Figure 3, red). All except 1 (*mni1*) of these 31 mutants were also long-lived when assayed as individual cultures: growth was detected in the higher dilutions at 9 wk (where wild-type cells did not grow), and even after 24 and 32 wk in some cases. Note that in the quantitative assay of Figure 1, wild-type cells largely remained viable for 9 wk, whereas in the qualitative assay of Figure 3 wild-type cells were largely dead at 9 wk. In the quantitative assay, cells were plated singly on rich media, whereas in the qualitative assay cells were spotted at high density. We speculate that long-term quiescent cells can revive better without competition by neighboring cells.

We verified that the correct genes were deleted for the 30 confirmed long-lived mutants by PCR testing for the predicted genomic-marker junctions created by gene deletion. Deletion for three mutants (SPBC8E4.02c, *rho2*, *gsk3*) could not be confirmed at both the 5′ and 3′ junctions, and for another three mutants (*vsp66*, *aps2*, SPACUNK4.16c) we could only confirm one of the two junctions. Although not conclusive, these results raise some doubts about the nature of these deletion mutants.

We also independently tested three random mutants (*hsp16*, SPBC839.03c, SPAC1F12.02c) with lifespan scores of <0.14, indicative of short-lived or sick mutants (Figure 3, green). All these mutants had largely lost viability at 9 wk and thus were somewhat shorter-lived than wild-type. In addition, five mutants (*ppk16*, *ubp5*, *ftp105*, *spt2*, *pnc1*) with lifespan scores between approximately 0.4 and 1.2 were randomly selected to test for any long-lived mutants, relative to wild-type, among mutants that did not make it onto the long-lived list but were among the top 15% with respect to lifespan score (Figure 3, orange). Notably, four of these five mutants (*ppk16*, *ubp5*, *ftp105*, *pnc1*) were longer-lived than wild-type in individual cultures. This result suggests that many mutants with lower lifespan scores can feature longer CLS compared with wild-type. Finally, we also tested the two mutants *pka1* and *git3*, for which no lifespan scores could be computed but that exhibit extended lifespan with limiting glucose during stationary phase (Roux *et al.* 2006). Both of these mutants also exhibited extended lifespan in our CLS assay (Figure 3, blue).

### Comparison of CLS mutants to published data

In budding yeast, three CLS screens of either individual or pooled stationary-phase cultures after glucose exhaustion have been reported (using microarrays instead of sequencing to quantify barcodes) (Powers *et al.* 2006; Fabrizio *et al.* 2010; Matecic *et al.* 2010). None of the orthologs of our 48 long-lived mutants have been identified in these budding yeast screens, which might indicate differences between the two chronological aging models and/or the two organisms. Notably, however, only five mutants (*far7*, *mds3*, *dcw1*, *puf3*, *trk1*) have been identified in two of the budding yeast screens, and no mutant has been identified in all three screens (Fabrizio *et al.* 2010; Matecic *et al.* 2010; Powers *et al.* 2006). However, several types of genes identified here also exhibit pro-aging effects in other systems. Deletion of *tif213*, encoding a subunit of the translation initiation factor eIF2, results in extended lifespan in our screen, similar to budding yeast mutants deficient in translation initiation activities (Bitterman *et al.* 2002). Two other regulatory genes corresponding to long-lived mutants in our screen, *tfs1* and *spo4*, encode a transcription elongation factor and serine/threonine kinase, respectively, as do the long-lived worm mutants *SPT4* and *AKT1* (Hamilton *et al.* 2005). Tfs1 affects chromatin patterns via global RNA polymerase II transcription (Reyes-Turcu *et al.* 2011). The histone methyltransferase Set9 exhibits pro-aging effects both in our system and in worms (Hamilton *et al.* 2005), as does the histone methyltransferase Set2 in budding yeast (Ryu *et al.* 2014). The predicted product of SPAC23D3.03c, a GTPase-activating protein whose absence extends lifespan in our system, is involved in vesicle-mediated transport. The PP2A-type phosphatase inhibitor Sds23 exhibits pro-aging effects both in our system and in budding yeast (Breslow *et al.* 2008). In contrast, Sds23 has also been reported to have anti-aging effects in fission yeast, because its overexpression extends lifespan of glucose-depleted cells during stationary phase (Roux *et al.* 2010). The *gsk3* mutant, whose gene encodes glycogen-synthase kinase 3, was among the most long-lived in our study, and inhibition of the orthologous *GSK-3* gene ameliorates a fly model of Alzheimer’s disease (Sofola *et al.* 2010). Gsk3 kinase promotes protein translation (Shin *et al.* 2011). There is a rich literature on protein translation and aging (Delaney *et al.* 2011) showing that reduced translation increases lifespan (Rallis *et al.* 2013; Selman *et al.* 2009).

Many of our long-lived mutants correspond to proteins associated with membranes. The following proteins are integral membrane proteins: Vps66, Gyp3, SPBC1198.07c, SPBC14C8.15, SPBC18H10.18c, SPCC594.02c, and SPBC30D10.09c. Three other proteins localize to the plasma membrane (Rho2, Ncs1 and Frp2), four proteins localize to the Golgi complex (Vps66, Fta5, SPAC23D3.03c and SPBC14C8.15), and eight proteins localize to the endoplasmic reticulum (Vps66, Fta5, SPBC1198.07C, SPCC1020.08, Frp2, SPCC594.02c, SPBC30D10.09c) (Matsuyama *et al.* 2006). Mutations in genes involved in the secretory pathway of the endoplasmic reticulum extend replicative lifespan in budding yeast (Labunskyy *et al.* 2014). SPACUNK4.16c is a predicted trehalose phosphate synthase, and the budding yeast *tsl1* mutant also exhibits extended chronological lifespan because of diminished trehalose synthesis (Kyryakov *et al.* 2012).

Several genes seem to have opposite effects on lifespan in budding and fission yeast. In budding yeast, mutants of the adenine biosynthesis pathway are long-lived (Matecic *et al.* 2010), whereas the corresponding mutants either decrease in abundance or are not detected in our screen (Table S2). The MutS mutants *msh2* and *msh6* are short-lived in budding yeast (Breslow *et al.* 2008), whereas the related mismatch-repair-defective *msh3* mutant was long-lived in our screen. The *gyp1* mutant, lacking a GTPase-activating protein, is short-lived in budding yeast (Giaever *et al.* 2002), whereas the *gyp3* and SPAC23D3.03c GTPase-activating protein mutants are long-lived in our screen. The *mkk1* MAP kinase kinase mutant is short-lived in budding yeast (Fabrizio *et al.* 2010), whereas the orthologous *pek1* mutant is long-lived in our screen. The *clg1* cyclin mutant is short-lived in budding yeast (Fabrizio *et al.* 2010), whereas the orthologous *mug80* cyclin mutant is long-lived in our screen. Finally, the *gpx2* glutathione peroxidase mutant is short-lived in budding yeast (Breslow *et al.* 2008), whereas the corresponding *gpx1* mutant is long-lived in our screen. These opposite effects might reflect differences between the two distantly related yeasts and/or between the two distinct chronological aging models involving glucose depletion in budding yeast and long-term quiescence on nitrogen depletion in fission yeast.

We also identified the following proteins not previously implicated in chronological aging: the Hht1 histone, the Abp2 DNA replication origin-binding protein, the Mrc1 replication-checkpoint mediator, the Cmk2 MAPK-activated protein kinase, the Vps66 acyltransferase (predicted), the Ncs1 calcium sensor, the Tfs1 transcription factor, and the Plb1 phospholipase, along with several orphans.

Notably, our screen did not identify any mutants that are known to be long-lived during stationary phase under glucose limitation (Rallis *et al.* 2014; Roux *et al.* 2010; Roux *et al.* 2009; Roux *et al.* 2006). The following fission yeast mutants, which are long-lived during stationary phase, were not detected in our screen due to low numbers of sequencing counts: *pka1* (cAMP-dependent protein kinase catalytic subunit) (Roux *et al.* 2006); *sck2* (S6 protein kinase) (Roux *et al.* 2006); and *git3* (glucose receptor) (Roux *et al.* 2009). We therefore independently tested *pka1* and *git3* mutants for longevity during quiescence. Notably, both mutants showed extended lifespan in individual cultures under nitrogen limitation (Figure 3). These results suggest that there is at least some similarity in the genetic basis underlying extended CLS in the two chronological aging models. The Pka1 and TOR pathways mainly control cellular responses to glucose and nitrogen, respectively, and are known to have common targets. For example, in budding yeast these two pathways converge on a single kinase to control entry to quiescence (Pedruzzi *et al.* 2003) or independently target processes vital for quiescence maintenance and survival (Stephan *et al.* 2009).

### Growth profiling of deletion mutants by Bar-seq

**Figure 4:**
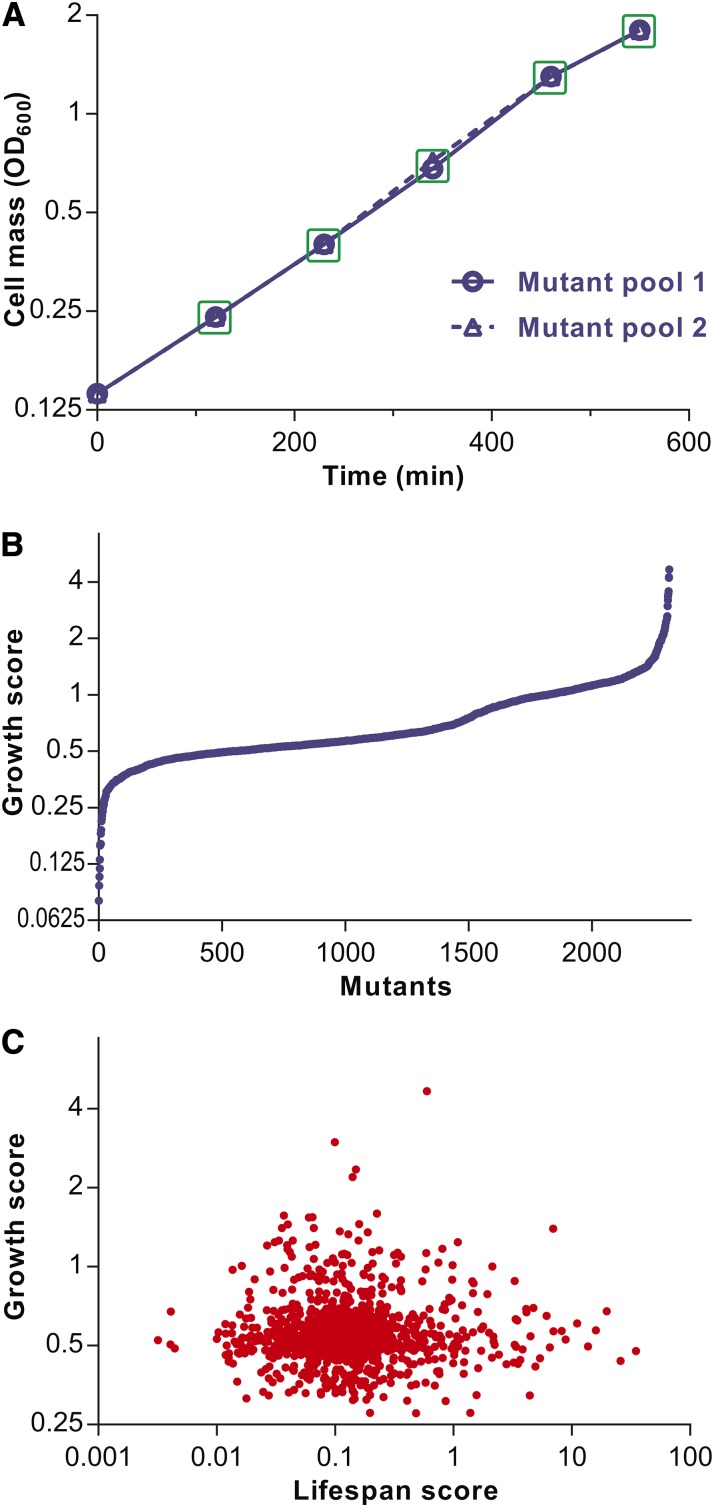
Growth profiling of deletion mutants and comparison to CLS. (A) Growth curves for two independent biological repeats of deletion mutant pool. The mutant pools were grown in EMM medium to exponential phase and diluted to OD_600_ 0.14 (timepoint 0). The OD_600_ was measured at 120, 230, 340, 460, and 550 min, and the pooled mutants were harvested at the same timepoints for Bar-seq analysis (green squares). (B) Plot of ordered growth scores for 2314 deletion mutants (see *Materials and Methods*). (C) Plot of growth scores *vs.* corresponding lifespan scores of 1193 mutants for which both scores could be determined. Growth and lifespan scores show no significant correlation (*r*_Pearson_ = −0.009; *P* = ∼0.75).

To our knowledge, the *S. pombe* deletion library has not been specifically analyzed for mutants with differences in proliferation under a standard, nonstress condition. We exploited the sensitive and efficient approach of parallel phenotyping to also compare the proliferation of the deletion mutants grown competitively in the same culture, followed by quantitative Bar-seq analyses. We performed two independent biological repeats of the mutant pool grown exponentially in EMM for 9 hr, with samples for Bar-seq analysis harvested at 5 timepoints between 120 and 550 min (Figure 4A). The sequence counts for each mutant in each sample are provided in Table S7. The sequence counts were normalized to the sequencing depth for each sample, and a growth score was determined for each mutant (see *Materials and Methods*). Growth scores could be computed for 2314 mutants; these scores are plotted in Figure 4B, with values provided in Table S8. The 231 most rapidly proliferating mutants are listed in Table S9, and the 231 most slowly proliferating mutants are listed in Table S10.

We looked for functional enrichments among the top 10% and 20% (231 and 463 genes, respectively) most slowly or most rapidly proliferating deletion mutants. The most slowly growing mutants were significantly enriched for the following categories (*P* < 0.02): conserved genes (Wood *et al.* 2002); core environmental stress response (CESR) genes that are downregulated during stress (Chen *et al.* 2003); ribosomal protein genes; top 500 most highly expressed genes (Wilhelm *et al.* 2008); and the GO category "macromolecule metabolism" (Blake *et al.* 2013), among others. These enrichments are expected for genes with important roles in cell growth. In budding yeast, mutants lacking ribosomal proteins have been identified as slow growers in a screen of individually growing mutants (Yoshikawa *et al.* 2011), and the expression of genes encoding ribosomal and macromolecule metabolic functions are induced during rapid growth (Castrillo *et al.* 2007; Brauer *et al.* 2008) . Also, in fission yeast ribosomal proteins are required for normal growth rate (Kim *et al.* 2010).

The most rapidly growing mutants were significantly enriched for the following categories (*P* < 0.02): genes without orthologs (Wood *et al.* 2012); CESR genes that are upregulated during stress (Chen *et al.* 2003); top 500 most lowly expressed genes (Wilhelm *et al.* 2008); and nitrogen-starvation response genes (Mata *et al.* 2002), among others. These categories are often the opposite of the categories for slowly growing mutants. Cells coordinate gene regulation with the growth rate to accommodate their physiological needs; under good conditions, cells grow rapidly but are stress-sensitive, whereas under poor conditions cells grow slowly or not at all (quiescence) but become stress-resistant (Lopez-Maury *et al.* 2008). This balance between cellular growth and maintenance is reflected in two large, antagonistically regulated gene expression programs in fission yeast, the CESR upregulated and downregulated genes (stress-related and growth-related genes, respectively) (Chen *et al.* 2003; Lopez-Maury *et al.* 2008; Pancaldi *et al.* 2010). It is interesting that deleting genes that are upregulated during stress or starvation leads to higher growth rates, which might reflect an evolutionary tradeoff between proliferation and stress protection. This finding also suggests that some of these stress-related genes are directly involved in inhibiting cell growth.

There are some indications that growth and lifespan are inversely correlated. For example, slow growth achieved by temperature manipulations results in extended lifespan of fish (Lee *et al.* 2013), and slow growth before entry into stationary phase is associated with longevity during stationary phase in fission yeast (Rallis *et al.* 2013). We therefore looked into the relationship between lifespan and growth in our data by plotting the growth scores and the corresponding lifespan scores for all mutants where both scores were available (Figure 4C). No significant inverse correlation was evident between lifespan and growth. Because many short-lived mutants might not be directly involved in chronological aging, we repeated the correlation analysis only for the 152 mutants with lifespan scores >0.4. Again, no significant inverse correlation between lifespan and growth was evident (*r*_Pearson_ = −0.03; *P* = ∼0.73). We conclude that slow growth is not associated with extended lifespan in our quiescence model, in contrast to the stationary-phase model. This difference could be rationalized by the extended time interval between the growth and quiescence phases (weeks or months), whereas the interval between the growth and stationary phases is much smaller (days). Quiescence might therefore represent a differentiated state that is not affected by the physiology during exponential growth before entry into quiescence. This finding further argues for the distinctiveness of the quiescence model, which might provide unique insights into chronological aging.

## Supplementary Material

Supporting Information
