# Supplementary material for: Parallel Profiling of Fission Yeast Deletion Mutants for Proliferation and for Lifespan During Long-Term Quiescence
Source: G3 (Bethesda). 2014 Dec 1;5(1):145–55. doi: 10.1534/g3.114.014415 (PMC4291465; doi:10.1534/g3.114.014415)
Supplement: Supporting Information [file supp_5_1_145__index.html]

Parallel Profiling of Fission Yeast Deletion Mutants for Proliferation and for Lifespan During Long-Term Quiescence — Parallel Profiling of Fission Yeast Deletion Mutants for Proliferation and for Lifespan During Long-Term Quiescence — Supporting Information 

# Parallel Profiling of Fission Yeast Deletion Mutants for Proliferation and for Lifespan During Long-Term Quiescence

## Supporting Information for Sideri *et al.*, 2015

**Files in this Data Supplement:**

- Table S1 - Barcodes for v2 Bioneer deletion mutants. (.xlsx, 108 KB)
- Table S2 - Sequence counts per mutant in each sample for standard CLS screen. (.xlsx, 357 KB)
- Table S3 - Lifespan scores from standard CLS screen. (.xlsx, 147 KB)
- Table S4 - 10% of mutants with lowest lifespan scores. (.xlsx, 32 KB)
- Table S5 - Sequence counts per mutant in each sample for re-growth CLS screen. (.xlsx, 379 KB)
- Table S6 - Long-lived mutants unique for re-growth CLS screen. (.xlsx, 14 KB)
- Table S7 - Counts per mutant in each sample for proliferation screen. (.xlsx, 374 KB)
- Table S8 - Growth scores from proliferation screen. (.xlsx, 156 KB)
- Table S9 - Top-10% most rapidly proliferating mutants in pool. (.xlsx, 22 KB)
- Table S10 - Top-10% most slowly proliferating mutants in pool. (.xlsx, 22 KB)
